# Predictors of Occurrence and 30-Day Mortality for Co-Infection of Carbapenem-Resistant *Klebsiella pneumoniae* and Carbapenem-Resistant *Acinetobacter baumannii*

**DOI:** 10.3389/fcimb.2022.919414

**Published:** 2022-06-20

**Authors:** Dongmei Lv, Yan Zuo, Yuerong Wang, Zhongxin Wang, Yuanhong Xu

**Affiliations:** Department of Clinical Laboratory, the First Affiliated Hospital of Anhui Medical University, Hefei, China

**Keywords:** co-infection, carbapenem-resistant *Klebsiella pneumoniae*, carbapenem-resistant *Acinetobacter baumannii*, risk factors, mortality, prognosis

## Abstract

**Background:**

The phenomenon of co-infection with multiple carbapenem-resistant bacteria is growing, which pose a great challenge for infection control and treatment. This study aimed to analyze predictors of occurrence and 30-day mortality for carbapenem-resistant *Klebsiella pneumoniae* and carbapenem-resistant *Acinetobacter baumannii* co-infection.

**Methods:**

From June 2018 to June 2021, clinical data of 103 patients co-infected with carbapenem-resistant *Acinetobacter baumannii* (CRAB) and carbapenem-resistant *Klebsiella pneumoniae* (CRKP) were collected from a tertiary teaching hospital in Anhui Province, China. The clinical characteristics and predictors of mortality were analyzed. Meanwhile, the bacterial isolates were characterized for drug susceptibility, multi-locus sequence typing, and drug resistance genes.

**Results:**

The multivariate analysis revealed that fiberoptic bronchoscopy (*p* = 0.005, OR=2.72), repeat transfusions (*p* = 0.008, OR= 2.23) and exposure to tigecycline (*p* = 0.002, OR = 6.58) were independent risk factors for CRKP and CRAB co-infection. Neutrophil ≥11.9*10^9^ (*p* = 0.035, adjusted HR = 3.12) and C-reactive protein ≥ 149 mg/L (*p* = 0.009, adjusted HR = 4.41) were found associated with 30-day mortality. Combined neutrophil with C-reactive protein could predict 30-day mortality, of which AUC value was 0.791 (95%CI: 0.661-0.921). KPC (46/51, 90.2%) was the most common carbapenemase in CRKP. 33 isolates of CRKP belong to *ST11* (33/51, 64.7%), and three new ST types *ST5882, ST5883, ST5885* were detected.

**Conclusions:**

Invasive operations and antibiotics exposure can lead to CRKP and CRAB co-infection. Combined neutrophil with C-reactive protein could predict 30-day mortality.

## Introduction

ESKAPE (Enterococcus faecalis, Staphylococcus aureus, Klebsiella pneumoniae, Acinetobacter baumannii, Pseudomonas aeruginosa, Enterobacter species) are major pathogens of hospital-acquired infections, and its increased drug resistance and widespread transmission have posed a serious threat to the health of hospitalized patients in recent years (De Oliveira et al., 2020). Increasing rates of drug resistance not only limit treatment options but also increase patient mortality and adverse outcomes (Nordmann and Poirel, 2014). Resistance not only have been increasing but also pandrug resistant Gram-negative pathogens (predominantly A. baumannii, K. pneumoniae and P. aeruginosa) have been increasingly reported worldwide and are associated with high mortality (Karakonstantis et al., 2020; Karakonstantis et al., 2020). According to the China Antimicrobial Surveillance Network (Hu et al., 2018; Hu et al., 2019), carbapenem resistance of K. pneumoniae has increased significantly from 4 to 25% between 2008 and 2021, while the resistance rate of A. baumanni is increasing each year reaching >70% in the last five years. The detection rate of K. pneumoniae in our region is not the highest, but the resistance rate to carbapenems has increased most rapidly in recent years, so it is of the greatest concern.

Carbapenem-resistant gram-negative bacteria infections increase the difficulty of treatment and lead to poor prognosis, which has been demonstrated in many studies (Marchaim et al., 2011). Therefore, the impact of multiple carbapenem-resistant bacterial co-infections on treatment and prognosis may be more complex. In the routine surveillance of bacterial infections, we found that CRKP and CRAB had the highest co-detection rate in our hospital. There are limited etiology and prognosis studies related to co-infection of CRKP and CRAB may due to the difficulty of searching cases of co-infection (Karakonstantis and Kritsotakis, 2021). The correlation between co-infection/colonization and multidrug resistance (MDR) may be related to the transfer of plasmid carrying resistance genes between multidrug-resistant bacteria, which needs serious attention. Quorum sensing is a mechanism for regulating the behavior of bacterial populations that has recently received increasing attention, which confers an increased phenotypic or behavioral resistance to different stress factors, including host defense mechanisms and antibiotics (Lazar et al., 2021), so it is worthwhile to consider how the two co-infected bacteria break the barrier to coexist and whether there is a link between them and hosts’ immunity. The limited availability of treatment may accelerate the disease progression and mortality from co-infection of multi-drug resistant bacteria (Marchaim et al., 2012). Therefore, there is an urgent need to investigate the clinic-etiology characteristics and prognosis of co-infection.

Therefore, this study was aimed to systematically analyze predictors of occurrence and molecular epidemiology for CRKP and CRAB co-infection. We also established a prognosis model of 30-day mortality for co-infection patients. Additionally, the treatment schemes in the retrospective data were especially analyzed.

## Methods

### Study Design and Patient Data

From June 1, 2018, to June 1, 2021, we collected *K. pneumoniae* and *A. baumanni* positive cases from a large tertiary hospital with 4800 beds in Hefei, Anhui Province. In this work, we focus on a subset of patients co-infected with multiple carbapenem-resistant bacteria. Therefore, co-infected candidates are defined based on the detection of ≥2 carbapenem-resistant bacteria in the same sample during hospitalization. The diagnosis of infection rather than colonization was confirmed by clinical signs and symptoms, imaging reports, and assessable clinical indicators (Mandell et al., 2007; Hooton et al., 2010).

To analyze the risk factors of CRKP-CRAB co-infection, this study set patients infected with only CRKP or CRAB as the two control groups. That is, three subgroups were identified: patients co-infected with CRKP and CRAB (case group), patients infected with CRKP alone (control group A), and patients infected with CRAB alone (control group B). The case and control groups were matched by age (± 3 years), gender, source of specimens, and ward.

The clinical data were obtained from the electronic medical system of the hospital. The clinical and epidemiological parameters included: age, gender, ward, length of hospitalization, treatment process (ICU admission, invasive surgery, and equipment), infection site, antibiotics exposure, baseline diseases, and outcomes. For the analysis of the risk factors for co-infection incidence, the events had to occur before the two isolates were detected. In addition, exposure to antibiotics was defined as the use of any antibiotic that has been continuously used for >3 days before the sample being cultured. Based on clinical, radiological, and laboratory tests results, the outcome of infection was defined as effective (cured or improved) or ineffective treatment (stable or worsening) (Zuo et al., 2021). Mortality was defined as death within 180 days from diagnosis of infection (including in-hospital death and post-discharge follow-up). All laboratory variables from the blood were obtained on the day of bacterial culture detection. This study was approved by the Ethics Committee of the First Affiliated Hospital of Anhui Medical University.

### Bacterial Strains and Antibiotic Susceptibility Testing

Due to poor strains preservation, we recovered a total of 102 co-infected strains, including 51 of each CRKP and CRAB isolates. All isolates were stored at -80°C and re-cultured on Columbia sheep blood agar plates at 37°C. The strain identification was performed by matrix-assisted laser desorption/ionization time-of-flight mass spectrometry (MALDI-TOF MS, BioMérieux, France). The genomic DNA was extracted from all samples for 16S rRNA identification of bacterial species with care to avoid any possible contamination. The results of the antibiotic susceptibility test were described according to the guidelines issued by the European Committee on Antimicrobial Susceptibility Testing (EUCAST).

### Characterization of Resistance Genes

Carbapenemase genes (*bla_KPC_, bla_NDM_, bla_IMP_, bla_VIM_, bla_SME_, bla_GES_, bla_OXA-23_, bla_OXA-48_, bla_OXA-51_*) (Woodford et al., 2006; Endimiani et al., 2008) were detected by polymerase chain reaction (PCR). The bacterial DNA of isolates was extracted by boiling in 1×TE. The PCR products were separated by gel electrophoresis, and gel recovery was confirmed by gene sequencing (China Biotech) and nucleotide BLAST search in GenBank.

### MLST (Multi-Locus Sequence Typing)

*K. pneumoniae* housekeeping genes were referred from the MLST website (http://bigsdb.web.pasteur.fr/klebsiella/primers-used.html). 7 pairs of housekeeping genes (*gapA, infB, mdh, pgi, phoE, rpoB, tonB*) were amplified and the PCR products were sent to Shanghai Sangon Company for sequencing (Diancourt et al., 2005). The sequencing results were compared with the MLST database (https://bigsdb.pasteur.fr/klebsiella/klebsiella.html) to obtain the corresponding allele and ST types.

### Statistical Analysis

All data were statistically analyzed using SPSS 16.0. Quantitative data are expressed as mean ± standard deviation or median (interquartile range). The t-test was used to compare variables with normal distribution or a nonparametric test was used otherwise. Qualitative data are expressed as ratios and compared by chi-square or Fisher’s exact test. *P* < 0.05 denote statistical significance. The multivariate analysis was used to analyze single factors with *p* < 0.2 using a binary logistic regression model, and the results are expressed as odds ratio (OR) values ​​and 95% confidence intervals (95% CI). Risk factors were identified by univariable and multivariable Cox regression analyses for predicting mortality, and the results are expressed as adjusted Hazard ratio (HR) and 95% confidence intervals (95% CI). The nomogram was constructed using the rms package in R software. Stepwise regression analysis was used to screen the indicators and obtain the probability prediction model of the joint indicators, the prediction accuracy was evaluated using the area under the receiver operating characteristic (ROC) curves.

## Results

### Characteristics of Patients

**Figure 1:**
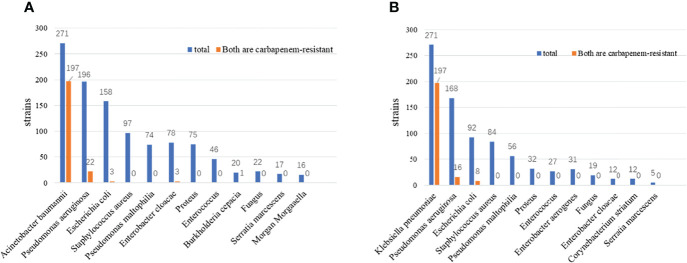
**(A)** Co-isolates in polymicrobial *Klebsiella pneumoniae* infections; **(B)** Co-isolates in polymicrobial *Acinetobacter baumannii* infections.

From June 2018 to June 2021, *K. pneumoniae* and *A. baumanni* positive culture detection were retrospectively collected, 1153 K*. pneumoniae* and 979 A*. baumanni* were co-detected with other bacteria (**Figure 1**). For *K. pneumoniae*, *A. baumanni* was the most common co-detected pathogen (271/1153, 23.5%), followed by *P. aeruginosa* (196/1153, 17.0%) and *E. coli* (158/1153, 13.7%). For *A. baumanni*, *K. pneumoniae* was the most common co-detected pathogen (271/979, 27.7%), followed by *P. aeruginosa* (168/979, 17.2%) and *E. coli* (92/979, 9.4%). Through the antibiotic susceptibility test, we found that 196 cases of *K. pneumoniae* and *A. baumanni* were both carbapenem-resistant in the co-detected samples. By excluding the patients with co-colonization, 103 cases were finally included. Among the 103 CRKP-CRAB co-infected patients, 66 (64.1%) were from ICU, 11 (10.7%) underwent neurosurgery, and 6 (5.8%) underwent cardiovascular Surgery. The most common infection site was the respiratory tract (87/103, 84.5%). A significantly higher proportion was noticed in males (76/103, 73.8%).

### Risk Factors in CRKP-CRAB Co-infected Patients

**Figure 2:**
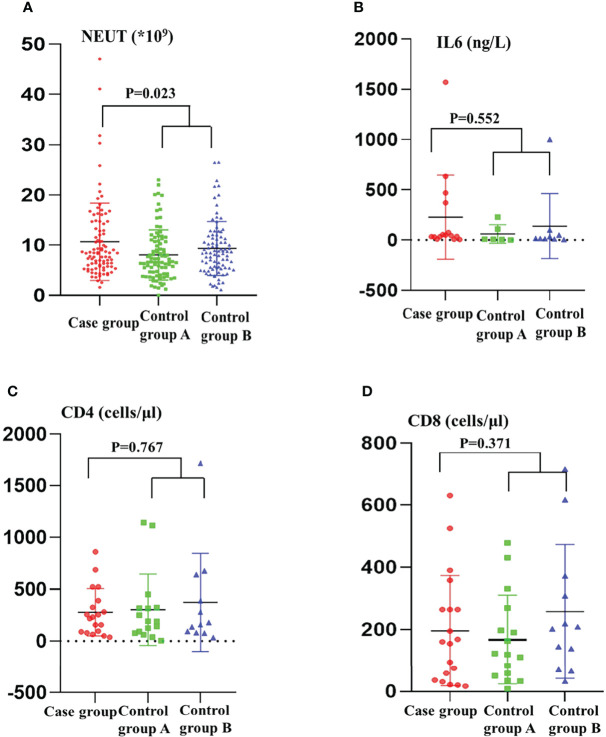
Blood test indexes in the case group and control groups. **(A)** NEUT; **(B)** IL6; **(C)** CD4; **(D)** CD8; NEUT, neutrophil; IL, Interleukin.

According to age, gender, specimen source, and ward 1:1:1 matching, a total of 94 patients were successfully matched out of 103 patients. The results of univariate analysis (**Table 1**) revealed that patients with previous extracorporeal membrane oxygenation (*p* = 0.007), fiberoptic bronchoscopy (*p* < 0.001), history of repeat transfusions (*p* < 0.001), exposure to polymyxin (*p* = 0.027) and tigecycline (*p* < 0.001) were more likely to suffer from co-infection. Also, the multivariate analysis (**Table 1**) shows that receiving fiberoptic bronchoscopy (*p* = 0.005, OR=2.72), repeat transfusions (*p* = 0.008, OR = 2.23) or exposure to tigecycline (*p* = 0.002, OR = 6.58) were independent risk factors for co-infection. The days of hospitalization before infection was also compared and a significant difference was found between the case group and control groups (p < 0.001). Blood test results showed statistically significant differences in neutrophil count (*p* = 0.023), and we also focused on immunological indicators and found no statistical difference in IL6, CD4, and CD8 (**Figure 2**).

**Table 1:** Univariate analysis and multivariate analysis of risk factors for CRKP and CRAB co-infected patients (case group) compared to control patients with CRKP infection alone (control group A) and control patients with CRAB infection alone (control group B).

Variable	case group (n = 94) n (%)/median (IQR)	control group n (%)/median (IQR)		P value	Multivariate analysis		
		A (n=94)	B (n=94)		OR	95%CI for HR	P value
**Baseline disease**							
Diabetes	12 (12.8)	5 (5.3)	7 (7.4)	**0.169**			
Heart dysfunction	23 (24.5)	23 (24.5)	16 (17.0)	0.363			
Chronic liver diseases	17 (18.1)	9 (9.6)	12 (12.8)	0.225			
Chronic nephrosis	17 (18.1)	20 (21.3)	15 (16.0)	0.639			
Malignancy	7 (7.4)	7 (7.4)	13 (13.8)	0.229			
Fracture	13 (13.8)	9 (9.6)	9 (9.6)	0.560			
**Therapeutic processes before infection**							
Mechanical ventilation	71 (75.5)	56 (59.6)	76 (80.9)	0.003			
Peripheral arterial catheter	38 (40.4)	22 (23.4)	29 (30.9)	0.042			
Central venous catheter	49 (52.1)	38 (40.4)	49 (52.1)	0.179			
Surgery	49 (52.1)	49 (52.1)	50 (53.2)	0.986			
Stomach tube	13 (13.8)	4 (4.3)	16 (17.0)	0.018			
Urinary catheter	65 (69.1)	60 (63.8)	61 (64.9)	0.718			
CRRT	17 (18.1)	16 (17.0)	7 (7.4)	0.071			
ECMO	9 (9.6)	1 (1.1)	2 (2.1)	**0.007**			
Fiberoptic bronchoscopy	31 (33.0)	7 (7.4)	13 (13.8)	**<0.001**	2.72	1.35-5.47	0.005
History of repeat transfusions	69 (73.4)	43 (45.7)	43 (45.7)	**<0.001**	2.23	1.24-4.03	0.008
Hemodialysis and plasma exchange	9 (9.6)	5 (5.3)	5 (5.3)	0.405			
Total hospital stay before infection, days (median, IQR)	18 (2-99)	7 (1-35)	9 (1-37)	**<0.001**			
**Exposure to antibiotics before infection**							
Penicillins	45 (47.9)	38 (40.4)	44 (46.8)	0.540			
Cephalosporins	51 (54.3)	39 (41.5)	55 (58.5)	0.052			
β-lactam/β-lactamase inhibitor	67 (71.3)	52 (55.3)	58 (61.7)	**0.075**			
Carbapenems	33 (35.1)	25 (26.6)	21 (22.3)	**0.140**			
Aminoglycosides	8 (8.5)	6 (6.4)	2 (2.1)	0.156			
Fluoroquinolones	8 (8.5)	5 (5.7)	11 (11.7)	0.292			
Glycopepetides	37 (39.4)	28 (29.8)	24 (25.5)	**0.113**			
Polymyxin	6 (6.4)	2 (2.1)	0 (0.0)	**0.027**			
Tigecyclines	15 (16.0)	4 (4.3)	0 (0.0)	**<0.001**	6.58	1.97-22.02	0.002
**Outcomes**							
30-day mortality	30 (31.9)	18 (19.1)	15 (16.0)	**0.021**			
180-day mortality	35 (37.2)	22 (23.4)	15 (16.0)	**0.003**			
Total hospital stay, days (median, IQR)	35 (4-155)	19(3-150)	23 (4-78)	**<0.001**			
length of hospital stay after infection (median, IQR)	16 (1-110)	8 (1-148)	13 (1-69)	**<0.001**			
Bacterial removal	44 (46.8)	49 (52.1)	52 (55.3)	0.499			
Effective treatment	54 (57.4)	69 (73.4)	74 (78.7))	**0.004**			

Data are expressed as n (%) unless stated otherwise. Variables with p < 0.2 in the univariate analysis were considered for the multivariate model. Bolded P values indicate statistical differences between the case group and the two control groups.

CRKP, carbapenem-resistant Klebsiella pneumoniae; CRAB, Carbapenem-resistant Acinetobacter baumannii; IQR, interquartile range; CRRT, continuous renal replacement therapy; ECMO, extracorporeal membrane oxygenation; CI, confidence interval; OR, odds ratio.

### Outcomes

**Figure 3:**
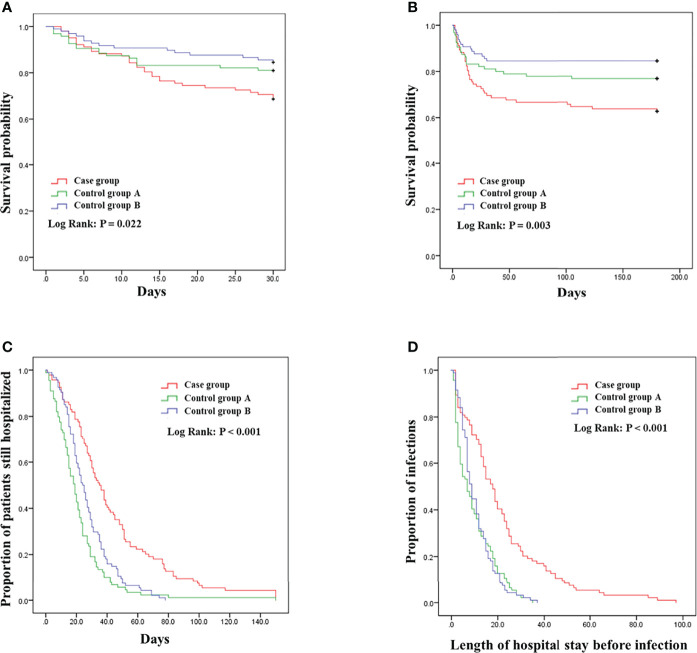
Kaplan–Meier survival plots, **(A)** 30-day mortality; **(B)** 180-day mortality; **(C)** Total day of hospitalization; **(D)** length of hospital stay before infection.

Compared with the control groups, co-infected patients had longer hospital stays (*p* < 0.001) and length of hospital stay after infection (*p* < 0.001), The14-, 30- and 180-day mortality rates were 21.3% (*p* = 0.086), 31.9% (*p* = 0.021) and 37.2% (*p* = 0.003) respectively (**Figure 3**), the median of overall mortality rate is 12 days. The case group had fewer effective treatment outcomes (*p* = 0.004).

### Risk Factors for 30-Day Mortality and Diagnosis Performance Analysis

**Figure 4:**
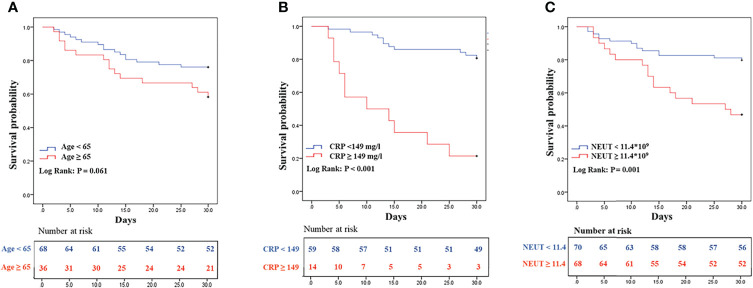
Kaplan–Meier survival plots, **(A)** Age; **(B)** CRP; **(C)** NEUT; NEUT, neutrophil; CRP, C-reactive protein.

**Figure 5:**
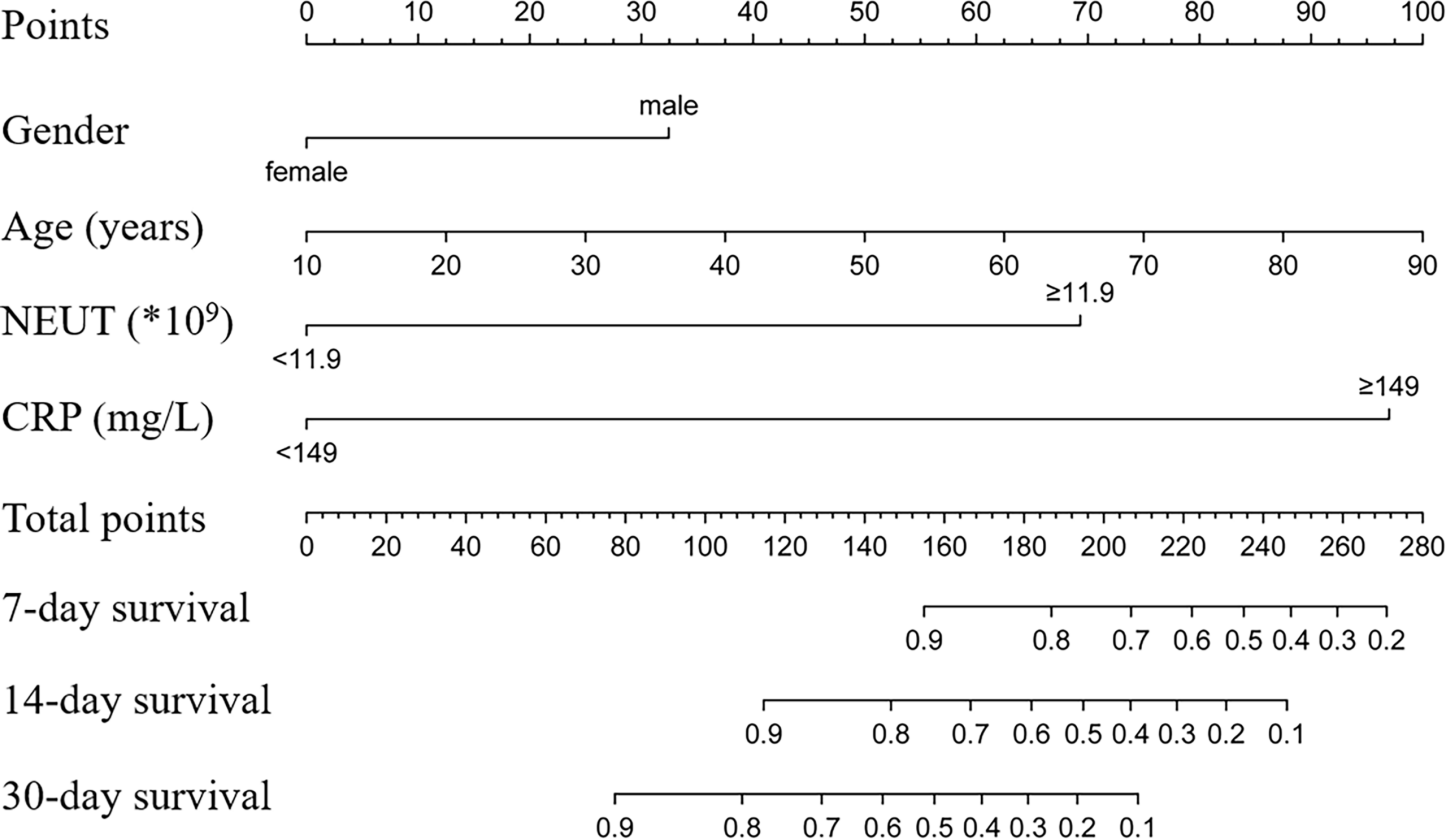
A predictive nomogram for predicting 7-, 14-, and 30-day mortality in patients with CRKP and CRAB co-infection. NEUT, neutrophil; CRP, C-reactive protein.

Out of 103 co-infected patients, 31 died and 72 survived in 30 days. The demographic details, clinical characteristics, and laboratory indicators are shown in **Table 2**, variables with P values < 0.05, and some clinically important variables were included in the Cox regression analysis model. Variables with P values < 0.1 in the univariate Cox regression analysis and other clinically important variables were included in the multivariate analysis (**Table 3**). Multivariate Cox analysis identified two variables for the final model, and the KM curves were plotted for the related variables (**Figure 4**). Neutrophil ≥11.9*10^9^ (adjusted HR = 3.12, 95% CI: 1.08-8.98, *p* = 0.035) and C-reactive protein ≥ 149 mg/L (adjusted HR = 4.41, 95% CI: 1.45-13.45, *p* = 0.009) were the independent factors for 30-day mortality. The above independent factors, along with age and gender, were used to establish the nomograms to predict the mortality in the patients with CRKP-CRAB co-infection (**Figure 5**).

**Table 2:** Univariate analysis of 30-day mortality of patients with CRKP and CRAB co-infection.

Variable	Total (n=103)	non-survivor (n=31)	survivor (n=72)	P value
**Demographic**				
Age	58 (17-88)	62 (27-84)	58 (17-88)	0.107
gender	76 (73.8)	25 (80.6)	51 (70.8)	0.299
Admission to an ICU	66 (64.1)	25 (80.6)	41 (56.9)	0.021
Total hospital stay, days (median, IQR)	35 (4-234)	23 (4-155)	40 (6-234)	0.007
History of hospital transfers	35 (34.0)	9 (29.0)	26 (36.1)	0.487
History of department transfers	61 (59.2)	17 (54.8)	44 (61.1)	0.552
**Baseline disease**				
Diabetes	13 (12.6)	5 (16.1)	8 (11.1)	0.482
Heart dysfunction	25 (24.3)	12 (38.7)	13 (18.1)	0.025
Neurologic disease	44 (42.7)	10 (32.3)	34 (47.2)	0.159
Chronic liver diseases	17 (16.5)	6 (19.4)	11 (15.3)	0.589
Chronic nephrosis	16 (15.5)	7 (22.6)	9 (12.5)	0.195
Malignancy	7 (6.8)	1 (3.2)	6 (8.3)	0.345
**Antimicrobial regimens**				
Number of anti-KP drugs, n (%)				
1	13 (16.7)	1 (4.8)	12 (21.1)	
≥2	65 (82.3)	20 (95.2)	45 (78.9)	0.087
Appropriate treatments within 3 days	78 (75.7)	21 (67.7)	57 (79.2)	0.215
Effective treatment	58 (74.4)	12 (57.1)	46 (80.7)	0.035
**Laboratory variables from blood, Mean ± SD**				
WBC (10^9^/L)	12.49 ± 8.33	15.25 ± 10.86	11.30 ± 6.71	0.073
NEUT (10^9^/L)	10.64 ± 7.55	13.64 ± 10.40	9.32 ± 5.46	0.038
LY (10^9^/L)	1.33 ± 2.20	1.71 ± 3.89	1.18 ± 0.69	0.466
NLR	13.16 ± 13.25	17.87 ± 14.41	11.09 ± 12.25	0.029
MONO (10^9^/L)	0.68 ± 0.77	0.57 ± 0.44	0.72 ± 0.87	0.35
Hb (g/L)	89 ± 22	84 ± 21	91 ± 23	0.132
PLT (10^9^/L)	208 ± 147	145 ± 144	236 ± 141	0.004
PT (sec)	15.5 ± 3.1	17.5 ± 3.8	14.5 ± 2.1	<0.001
APTT (sec)	41.7 ± 12.9	49.1 ± 12.7	37.8 ± 11.3	<0.001
D-dimer (mg/L)	6.4 ± 5.9	6.1 ± 5.5	6.6 ± 6.1	0.772
Albumin (g/L)	34.8 ± 5.8	33.9 ± 6.8	35.2 ± 5.4	0.331
A/G	1.54 ± 0.39	1.69 ± 0.44	1.48 ± 0.36	0.012
ALT (U/L)	85 ± 150	113 ± 249	72 ± 73	0.381
AST (U/L)	88 ± 135	124 ± 207	72 ± 83	0.191
AST/ALT	1.42 ± 1.56	1.69 ± 1.33	1.31 ± 1.51	0.203
LDH (U/L)	567 ± 804	775 ± 1166	473 ± 565	0.259
CRP (mg/L)	75.17 ± 74.6	122.07 ± 96.40	53.67 ± 50.12	0.004
Creatinine (umol/L)	98.1 ± 85.1	138.2 ± 98.7	81.8 ± 73.6	0.009
PCT (ng/mL)	6.08 ± 15.19	15.43 ± 24.26	1.65 ± 2.46	0.007
IL6 (ng/L)	242.72 ± 431.18	537.58 ± 640.68	78.90 ± 111.12	0.182
CD4 (cells/μL)	285 ± 224	258 ± 204	310 ± 247	0.613
CD8 (cells/μL)	184 ± 173	159 ± 150	207 ± 196	0.542
CD4/CD8	2.24 ± 1.64	2.29 ± 2.11	2.20 ± 0.99	0.907

Data are expressed as n (%) unless stated otherwise.

CRKP, carbapenem-resistant Klebsiella pneumoniae; CRAB, Carbapenem-resistant Acinetobacter baumannii; IQR, inter-quartile range; CRRT, continuous renal replacement therapy; ECMO, extracorporeal membrane oxygenation; WBC, white blood cell count; Hb, hemoglobin; PLT, platelet; NEUT, neutrophil; LY, lymphocyte; NLR, neutrophil-to-lymphocyte ratio; MONO, monocytes; A/G, albumin/globulin; ALT, alanine aminotransferase; AST, aspartate aminotransferase; LDH, Lactate dehydrogenase; PT, Prothrombin Time; APTT, Activated Partial Thromboplastin Time; PCT, procalcitonin; CRP, C-reactive protein; IL, Interleukin.

**Table 3:** Univariate and multivariate Cox regression analysis of risk factors for 30-day mortality.

Variable	Univariate analysis			Multivariate analysis		
	HR	95%CI for HR	P value	aHR^a^	95%CI for HR	P value
Sex, male vs female	1.61	0.66-3.93	0.295			
Age, year, ≥65 vs <65	1.90	0.94-3.85	0.074			
Heart dysfunction	2.21	1.07-4.56	0.031			
WBC, 10^9/^L, ≥14.3 vs <14.3	3.15	1.53-6.50	0.002			
NEUT, 10^9^/L, >11.9vs <11.9	3.37	1.63-6.96	0.001	3.12	1.08-8.98	0.035
NLR, ≥10.3vs <10.3	4.37	1.99-9.58	<0.001			
PLT, 10^9^/L, <75 vs ≥75	5.23	2.55-10.75	<0.001			
PT, ≥16 vs <16	4.44	2.06-9.56	<0.001			
APTT, ≥42 vs <42	4.97	2.19-11.26	<0.001			
A/G, ≥1.69 vs <1.69	2.85	1.39-5.85	0.004			
CRP, mg/L, ≥149 vs <149	8.34	3.48-19.96	<0.001	4.41	1.45-13.45	0.009
Creatinine, μmol/L, ≥97 vs <97	3.34	1.58-7.09	0.002			
PCT, ng/mL, ≥3 vs <3	4.72	2.20-10.16	<0.001			
Effective treatment	0.51	0.25-1.05	0.068			

^a^Adjusted hazard ratio for age and sex.

The thresholds for blood tests are based on the cut-off point of the ROC curve.

CI, confidence interval; HR, hazard ratio; WBC, white blood cell count; PLT, platelet; NEUT, neutrophil; NLR, neutrophil-to-lymphocyte ratio; A/G, albumin/globulin; PT, Prothrombin Time; APTT, Activated Partial Thromboplastin Time; PCT, procalcitonin; CRP, C-reactive protein.

**Figure 6:**
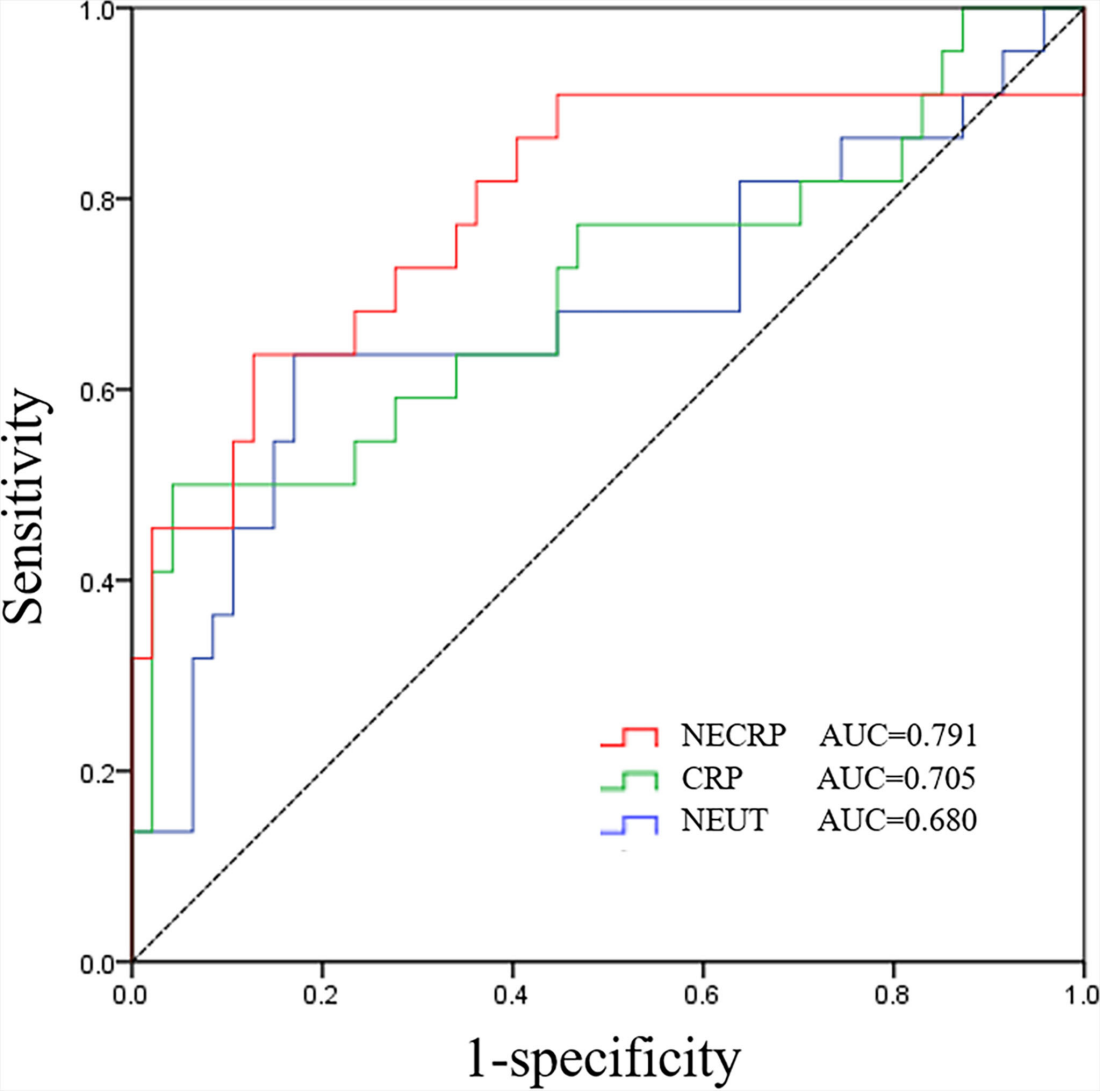
Combined model, neutrophil and C-reactive protein for predicting 30-day in CRKP-CRAB co-infection.

By the stepwise regression method, the prediction model of NEUT combined with CRP for predicting 30-day mortality was obtained: NECRP = 0.016 × NEUT + 0.002 × CRP − 0.037. The ROC curve (**Figure 6**) shows that the AUC value of the prediction model is 79.1% (95%CI: 0.661-0.921, *p* < 0.001). The combined variable equation predicts 30-day mortality in co-infected patients higher than the two variables alone.

### Antimicrobial Therapy

**Figure 7:**
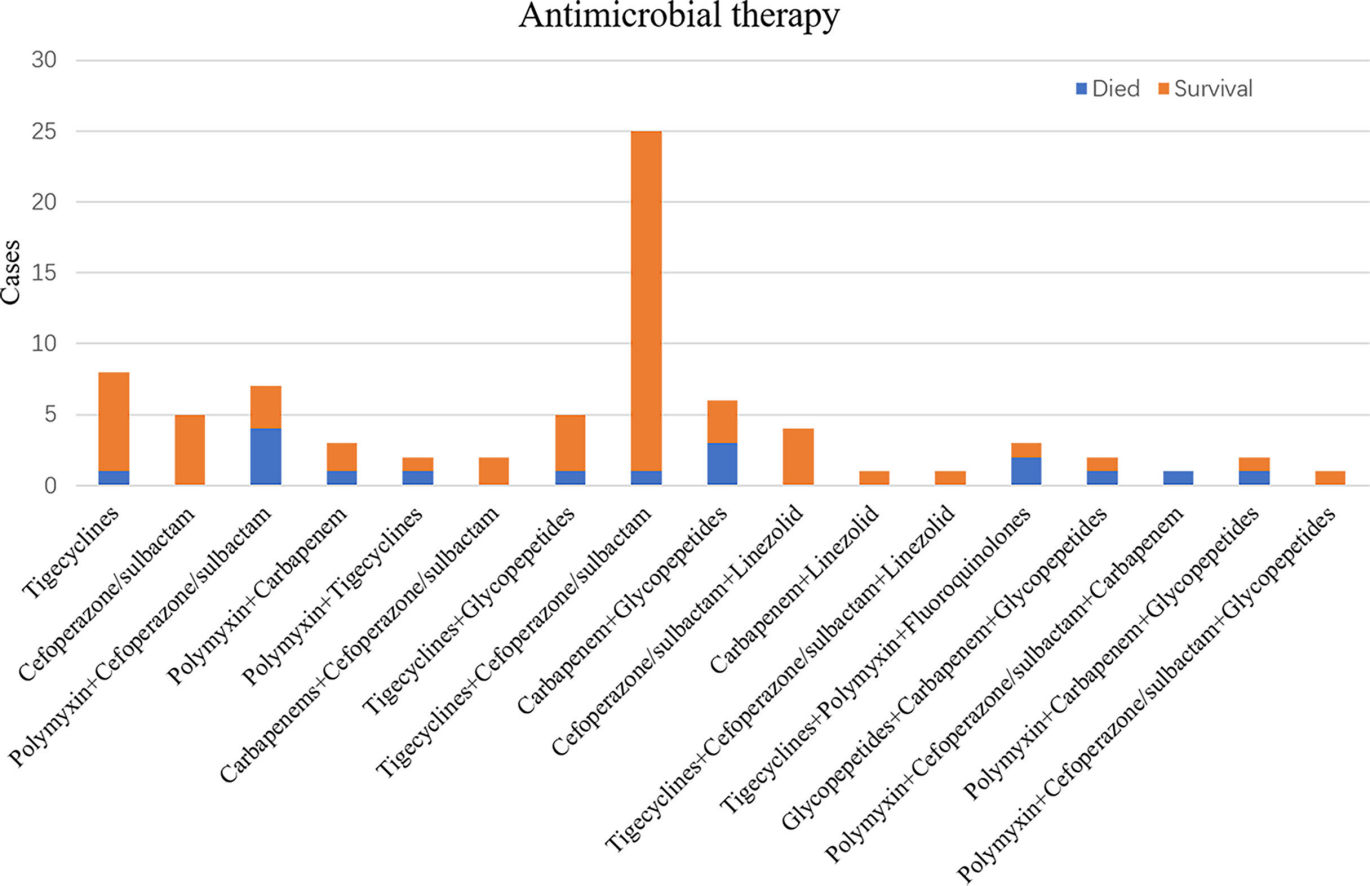
Antimicrobial therapy classification of 78 patients with co-infection treated with antimicrobial drugs and mortality.

Among the 103 patients in the test group, only 78 (75.7%) received timely antimicrobial treatment within three days (**Figure 7**), and the remaining did not receive antimicrobial therapy because of discharged, transferred, or died before the drug sensitivity results were reported. Among the 78 cases, 7.7% were treated with monotherapy, the mortality rate was 7.8%, which was lower than the combined treatment group. The monotherapy group mainly used tigecycline or cefoperazone/sulbactam with tigecycline as the main therapy. Combination therapy includes dual therapy and triple therapy, among which dual therapy is the main. Tigecycline combined with cefoperazone/sulbactam is the most common combination used by clinicians, with a low mortality rate of 4% compared to other types of combination therapy. Combination regimens centered on tigecycline and polymyxin, reflecting the fact that these two drugs have become the mainstay against carbapenem-resistant pathogenic bacteria.

### Characterization of Resistance Genes and MLST

For carbapenemase detection, we collected CRAB and CRKP from 51 patient cultures. *bla_KPC_* (46/51, 90.2%) was the most common carbapenemase in CRKP, followed by *bla_NDM_* (4/51, 7.8%). However, all CRAB isolates were positive for *bla_OXA-23_* and *bla_OXA-51_*.

MLST analysis revealed 10 sequence types among 51 K*. pneumoniae* strains, including three newly identified STs (*ST5882, ST5883*, and *ST5885*), of which 33 K*. pneumoniae* belong to *ST11* (33/51, 64.7%), the other types include *ST15* (8/51, 15.7%), *ST45* (2/51, 3.9%), *ST36* (2/51, 3.9%), *ST4267* (1/51, 2%), *ST412* (1/51, 2%), *ST17* (1/51, 2%), *ST5882* (1/51, 2%), *ST5883* (1/51, 2%), and *ST5885* (1/51, 2%).

## Discussion

Carbapenem-resistant bacterial infections are a pressing public health concern due to its rapid spread and the limited availability of treatment. Current treatment options include drug combinations such as polymyxin and tigecycline with other antibiotics. Ceftazidime/avibactam is also quite effective in the treatment of carbapenem-resistant Enterobacteriaceae (Zhong et al., 2018; Doi, 2019). However, antibiotic abuse, environmental transmission, quorum sensing and host adaptative response are increasing co-infection of carbapenem-resistance bacteria and further increase the difficulty of clinical treatment. Our study provides a reference for CRAB and CRKP co-infection.

From the overall review from June 2018 to June 2021 in our hospital, we found a significantly high prevalence of co-infections of CRAB and CRKP, although the detection rate of these two pathogens is lower than that of *E. coli* in this hospital. In a recent systematic review of co-isolates of the polymicrobial *A. baumanni*, *P. aeruginosa* and *K. pneumoniae* were shown to be common co-isolates of *A. baumanni*, in agreement with the results of our study (Karakonstantis et al., 2022). 64% of CRKP-CRAB co-infected patients were from ICU, airborne and contact transmission of drug-resistant bacteria in the ICU setting may lead to hospital-acquired infections or colonization. ICU patients usually undergo more invasive procedures requiring longer antibiotic therapy. All these factors may contribute to the emergence and spread of carbapenem-resistant bacteria (Petrosillo et al., 2013). The respiratory tract is the most common infection site. It is worth pondering to establish control strategies against ICU infection in the future (Qin et al., 2020).

In this study, we developed a multivariate model with two control groups. It was clear that fiberoptic bronchoscopy, repeat transfusions, and exposure to tigecycline were independent risk factors for CRKP and CRAB co-infection. So far, there are only a few literature reports about risk factors for bacterial co-infection (Marchaim et al., 2012; Mammina et al., 2013). Invasive surgery can lead to carbapenem-resistant bacterial infection has become a well-known phenomenon (Zuo et al., 2021), however, whether it leads to co-infection was investigated in this study. Bronchoscopy is one of the standard methods to diagnose respiratory diseases. Studies have shown that *A. baumannii* and *K. pneumoniae* readily colonizes the surfaces of medical devices, especially tracheal tubes, by forming biofilms (Chung, 2016; Roy et al., 2022). Bringing colonized bacteria into the lesion during the examination and subsequently causing infection (Sato et al., 2020). Therefore, it is essential to enhance operational hygiene and infection control measures for aseptic procedures (Manandhar et al., 2021). Blood transfusions are commonly used to treat patients with acute blood loss during surgery and hypoproteinemia, who are relatively immune-compromised (Zhai et al., 2022). Additionally, adverse effects from transfusions are common. Therefore, we speculate that on the one hand, the blood products themselves act as a source of contamination leading to infection, and on the other hand, changes in the patient’s immune system destroy the patient’s defense barrier further allowing pathogens in the environment to take advantage, thereby increasing the risk of co-infection (Cata et al., 2013). Exposure to tigecycline also was a risk factor for co-infection. Due to the growing bacterial resistance, tigecycline has become one of the few options to treat serious carbapenem-resistant bacterial infections. Whereas, the adverse effects of treatment, as well as antibiotic pressure, may alter the microflora and the resistance of co-infected bacteria in patients, increase the predominance of certain drug-resistant bacteria (Li et al., 2020), which further provides conditions for the mutation and resistance evolution of gram-negative bacteria. Studies have shown that high doses of tigecycline, such as 100 mg every 12 hours, are more effective in treating multidrug-resistant gram-negative bacteria, and that lower doses may not only be insufficient to treat bacterial infections but also increase antibiotic pressure leading to the growth of dominant flora (Zhou et al., 2021). Of concern is the gradual emergence of tigecycline-resistant *K. pneumoniae* and *A. baumannii* due to the widespread use of tigecycline, which undoubtedly increases the challenge of antibiotic therapy, making optimization of tigecycline treatment regimens urgent for clinicians (Goodarzi et al., 2021; Sun et al., 2022). We also focused on the differences in blood tests between the three groups, the results of host immune indicators indicate that the co-infection response is mainly countered by neutrophils, which have no effect on T cell proliferation. Of course, it requires a larger sample size for a more comprehensive analysis.

Patients in the case group had a higher length of stay and mortality compared to the control group. Prolonged hospitalization is a well-known risk factor for antibiotic-resistant bacterial infections due to more invasive procedures and the use of multiple antibiotics (Cohen et al., 2006). CRKP co-colonization/infection with non-fermenting gram-negative bacteria significantly increases the mortality risk has been reported (Marchaim et al., 2011; Marchaim et al., 2012). Significant increase in length of hospitalization before infection in co-infected patients, a stronger indication that long hospital stays increase the risk of co-infection. There were fewer effective treatment outcomes in the case group, suggesting that the current common clinical drugs for carbapenem-resistant bacterial infections are less effective against co-infecting bacteria, and that clinicians need to continue to improve.

In this study, the ST11 type CRKP that produces KPC carbapenemase is the main type, which is consistent with most national studies (Chen et al., 2014; Gu et al., 2018; Meng et al., 2019). *bla_NDM_* was also detected. The two carbapenem-resistant bacteria did not reveal the same resistance gene, suggesting that there may not be a horizontal transfer of resistance plasmids between the two, but more likely due to antibiotic pressure, microbial dysbiosis in patients and the evolution of the bacteria resulting in coexistence in the environment. In addition, we identified three new ST subtypes that had not been previously identified in related studies, suggesting that co-infected bacteria have special molecular biological characteristics and there are novel CRKP mutants in our hospital.

This study indicated the Neutrophil and C-reactive protein are predictors of 30-day mortality. Neutrophils are an important component of the immune system and are the first line of defense against bacterial infections. They rapidly recruit and phagocytose to kill pathogenic microorganisms after bacterial infections (Kobayashi et al., 2018), so they will show an increase after infection. CRP is one of the most distinctive indicators of inflammation, involvement in the inflammatory response *in vivo* through complement activation and receptor-mediated modalities, exhibiting different levels at different time points of pathogenic infection (Karasahin et al., 2018; Luo et al., 2020). The normal range of CRP is 0-10mg/L. Because of its high sensitivity and is susceptible to multiple factors such as age gender underlying disease, it changes significantly with the severity of the disease and the level of inflammatory response in patients. This study refers to the cut-off point of the receiver operating characteristic curve, with 149mg/L as the threshold better reflecting the prognostic value of high levels of CRP. The area under the ROC curve for the combined diagnosis of NEUT and CRP was 0.791, which was higher than the AUC for the two variables alone, indicating that the combined diagnosis had a better predictive value for prognosis. It is worth emphasizing that although the association of 30-day mortality with neutrophils and CRP has been demonstrated statistically, its value for application in clinical practice remains to be evaluated. Collecting queues for model validation is also our future endeavor.

In this study, we discussed the effects of different drug treatments particularly, hoping to give clinical practitioners a reference. Only 75.7% of patients received timely antimicrobial therapy and the mortality rate after treatment account for 26.9%, a large number of patients were treated with multiple drug combinations. The mortality rate of monotherapy was lower than that of combined therapy, which is an unexpected finding. The reasons for this phenomenon may be related to adverse drug reactions. The combined therapy greatly increases adverse reactions, especially renal toxicity, which can lead to poor prognosis for patients with renal failure. On the other hand, studies have shown that polymyxins are associated with higher mortality. Without polymyxins in our monotherapy instead the combination therapy contains quite a few combinations of polymyxins, and our patients may not be receiving the optimal dose for their use (de Oliveira et al., 2015). In addition, a number of studies have found that combination therapy did not reduce mortality in gram-negative infections (Amat et al., 2018; Alrahmany et al., 2021). The most common drug combination was tigecycline + cefoperazone/sulbactam, which also showed a lower mortality rate, other drug combinations were not informative due to the small number of cases. Clinicians should adjust and improve the antibiotic regimens in time according to different patient conditions to achieve better efficacy.

Nonetheless, the present study has some limitations, the first being the bias introduced by the single-center retrospective study itself. Therefore, we need a multicenter prospective study to eliminate the bias. In addition, the limited sample size of this study prevented a more systematic study of the effects of antimicrobial therapy. Death models also require external validation. and finally, we can use sequencing technology to further explore the causes of co-infection and the mechanisms of interaction with the host in the future.

## Conclusion

In summary, this is the major study in China that examined the risk factors and prognosis of CRAB-CRKP co-infection. The major risk factors include fiberoptic bronchoscopy, history of repeat transfusions and exposure to tigecycline. Co-infected patients have higher mortality rates and longer hospital stays. In addition, patients Neutrophil ≥11.9*10^9^ and C-reactive protein ≥ 149 mg/L were predictors of 30-day mortality, the combined diagnosis of the two has a better predictive value. The treatment of patients with co-infection needs to be adjusted timely by clinicians according to the patient’s situation. In conclusion, clinicians should improve their treatment protocols for multi-bacterial infections, as well as strengthen prevention and control measures of nosocomial infections.

## Data Availability Statement

The raw data supporting the conclusions of this article will be made available by the authors, without undue reservation.

## Ethics Statement

This study was reviewed and approved by the Ethics Committee of the First Affiliated Hospital of Anhui Medical University. The written informed consent was obtained from patients in accordance with the Declaration of Helsinki.

## Author Contributions

DL and YZ: concept and writing of the manuscript. DL and YW: data processing and software. ZW and YX: review and editing. All authors contributed to the article and approved the submitted version.

## Funding

This work was financially supported by Anhui Natural Science Foundation (grant number: 9021138201) and the Scientific Research Project of Universities in Anhui Province (grant number: KJ2020A0170).

## Conflict of Interest

The authors declare that the research was conducted in the absence of any commercial or financial relationships that could be construed as a potential conflict of interest.

## Publisher’s Note

All claims expressed in this article are solely those of the authors and do not necessarily represent those of their affiliated organizations, or those of the publisher, the editors and the reviewers. Any product that may be evaluated in this article, or claim that may be made by its manufacturer, is not guaranteed or endorsed by the publisher.
